# Blockade of HCN2 Channels Provides Neuroprotection Against Ischemic Injury *via* Accelerating Autophagic Degradation in Hippocampal Neurons

**DOI:** 10.1007/s12264-020-00513-7

**Published:** 2020-06-10

**Authors:** Cheng Chen, Li Liu, Ya-Qiao Shu, Ping Jing, Yun Lu, Xiao-Xue Zhang, Xian-Gang Zong, Lian-Jun Guo, Chang-Jun Li

**Affiliations:** 1 Department of Pharmacology, School of Basic Medical Sciences, Tongji Medical College, Huazhong University of Science and Technology, Wuhan, 430030 China; 2 Department of Neurology, The Central Hospital of Wuhan, Tongji Medical College, Huazhong University of Science and Technology, Wuhan, 430030 China; 3 Office of Academic Research, The Central Hospital of Wuhan, Tongji Medical College, Huazhong University of Science and Technology, Wuhan, 430030 China; 4 Department of Pharmacy, Zhongnan Hospital of Wuhan University, Wuhan, 430030 China; 5 Department of Clinical Laboratory, Wuhan PuAi Hospital, Wuhan, 430033 China; 6 Key Laboratory of Drug Target Research and Pharmacodynamic Evaluation, Wuhan, 430030 China; 7 Center for Integrated Protein Science and Zentrum für Pharmaforschung, Department Pharmazie, Ludwig-Maximilians-Universität München, 80539 Munich, Germany

**Keywords:** HCN2 channel, Autophagy, Neuroprotection, Oxygen-glucose deprivation/reperfusion, Transient global cerebral ischemia

## Abstract

In the central nervous system, hyperpolarization-activated cyclic nucleotide-gated (HCN) channels are essential to maintain normal neuronal function. Recent studies have shown that HCN channels may be involved in the pathological process of ischemic brain injury, but the mechanisms remain unclear. Autophagy is activated in cerebral ischemia, but its role in cell death/survival remains controversial. In this study, our results showed that the HCN channel blocker ZD7288 remarkably decreased the percentage of apoptotic neurons and corrected the excessive autophagy induced by oxygen-glucose deprivation followed by reperfusion (OGD/R) in hippocampal HT22 neurons. Furthermore, in the OGD/R group, p-mTOR, p-ULK1 (Ser^757^), and p62 were significantly decreased, while p-ULK1 (Ser^317^), atg5, and beclin1 were remarkably increased. ZD7288 did not change the expression of p-ULK1 (Ser^757^), ULK1 (Ser^317^), p62, Beclin1, and atg5, which are involved in regulating autophagosome formation. Besides, we found that OGD/R induced a significant increase in Cathepsin D expression, but not LAMP-1. Treatment with ZD7288 at 10 μmol/L in the OGD/R group did not change the expression of cathepsin D and LAMP-1. However, chloroquine (CQ), which decreases autophagosome-lysosome fusion, eliminated the correction of excessive autophagy and neuroprotection by ZD7288. Besides, shRNA knockdown of HCN2 channels significantly reduced the accumulation of LC3-II and increased neuron survival in the OGD/R and transient global cerebral ischemia (TGCI) models, and CQ also eliminated the effects of HCN2-shRNA. Furthermore, we found that the percentage of LC3-positive puncta that co-localized with LAMP-1-positive lysosomes decreased in Con-shRNA-transfected HT22 neurons exposed to OGD/R or CQ. In HCN2-shRNA-transfected HT22 neurons, the percentage of LC3-positive puncta that co-localized with LAMP-1-positive lysosomes increased under OGD/R; however, the percentage was significantly decreased by the addition of CQ to HCN2-shRNA-transfected HT22 neurons. The present results demonstrated that blockade of HCN2 channels provides neuroprotection against OGD/R and TGCI by accelerating autophagic degradation attributable to the promotion of autophagosome and lysosome fusion.

## Introduction

Stroke is a common cerebrovascular disease accompanied by high mortality and morbidity, imposing enormous social and economic burdens [1]. According to an epidemiological survey, cerebral ischemia accounts for approximately 87% of strokes [2]. Restoring blood flow to the affected area as early as possible is considered to be the most effective treatment of cerebral ischemia [3, 4]. However, restoration of the blood supply further aggravates ischemia-induced brain damage, which is termed ischemia/reperfusion (I/R) injury [5]. The pathophysiological mechanisms of cerebral I/R injury are not fully clarified yet, and more effective therapeutic strategies for cerebral ischemia still need to be explored.

Autophagy is a crucial lysosomal process for the degradation of damaged or unnecessary intracellular organelles, proteins, and other cell components to maintain homeostasis, and is involved in various basic physiological processes such as quality control of proteins and organelles, immunity, development, and differentiation [6]. Appropriate autophagic activity is necessary for the maintenance of normal intracellular homeostasis [7] and survival under certain environmental stress conditions [8]. However, excessive autophagy induced by various stressors may contribute to cell death [9, 10]. Increasing evidence has indicated that, although autophagy is activated in cerebral ischemia, its role in cell death or survival remains controversial [11]. Some studies have reported that autophagy protects against the neuronal injury induced by cerebral ischemia [12, 13]. Conversely, other studies have shown that the inhibition of autophagy protects against cerebral ischemia injury [14–17]. Thus, the role of autophagy in cerebral ischemia injury needs further investigation.

Hyperpolarization-activated cyclic nucleotide-gated (HCN) channels are encoded by a family of HCN1-4 genes and have four isoforms. HCN1 and HCN2 are abundantly expressed in the rodent hippocampus [18], but HCN3 and HCN4 are expressed at very low levels [19]. HCN channels are activated when the cell membrane is hyperpolarized and permeate K^+^ and Na^+^ ions to generate the inward *I*_h_ current in the nervous system [19]. *I*_h_ plays important roles in the stability of neuronal resting membrane potential [20], neuronal rhythmicity [19, 21], the periodicity of network oscillations [22], dendritic integration [23, 24], synaptic plasticity [25, 26], and neurotransmitter release [27, 28]. In general, *I*_h_ currents are activated at potentials negative to − 50 mV to − 60 mV [19]. In CA1 pyramidal cells, the mean resting membrane potential is − 64 ± 2 mV, and oxygen–glucose deprivation (OGD) produces an initial hyperpolarization that ranges from 5 mV to 20 mV within 5 min after exposure [29], when *I*_h_ channels are activated. In a previous study, we have shown that the surface expression of HCN1 and HCN2 is dysregulated in the rat hippocampal CA1 area under chronic cerebral hypoperfusion [30]. Pavel Honsa *et al*. have reported that the expression of HCN channels is increased in reactive astrocytes following focal cerebral ischemia [31]. However, the biological effects of changes in HCN channel activation and expression during cerebral ischemia and possible mechanisms have yet to be revealed.

In this study, we investigated the regulation of autophagy by HCN channels and its effect on the neuronal ischemic injury induced by OGD followed by reperfusion (OGD/R) and transient global cerebral ischemia (TGCI).

## Materials and Methods

### Chemicals

Chloroquine (CQ) was from Sigma (St. Louis, MO); ZD7288 was from Tocris Cookson (Bristol, UK); and Dulbecco’s modified Eagle’s medium (DMEM) was from Gibco Invitrogen (Grand Island, NY). ZD7288 and CQ were prepared as stock solutions in sterile water and stored at − 20 °C away from light. Subsequent solutions of specific concentrations (ZD7288: 1 μmol/L, 5 μmol/L, 10 μmol/L, and 20 μmol/L; CQ: 50 μmol/L) were made in culture medium or sterile water.

### Cell Culture and OGD/R

The mouse HT22 hippocampal neuronal cell line was from Jennio Biotech Co., Ltd (Guangzhou, China). HT22 neurons were cultured in DMEM supplemented with 10% (*v*/*v*) fetal bovine serum, 100 U/mL penicillin, and 100 µg/mL streptomycin in a humidified incubator under 5% (*v*/*v*) CO_2_ at 37 °C.

The method of OGD induction was described in detail in our previous publication [32], and its duration was 2 h, 4 h, 6 h, 8 h, or 12 h. At the end of these periods, cultures were returned to oxygenated, glucose-containing DMEM under normoxic conditions for 12 h (reperfusion). ZD7288 or CQ was added to the medium 2 h prior to OGD and left until the end of reperfusion.

### Lentiviral Transduction

Small-hairpin (sh)RNA nucleotide (CGTGGTTTCGGATACTTTCTTCCTCA) against the *HCN2* gene (NM_053684) and a control sequence (TTCTCCGAACGTGTCACGT) were selected in accordance with previous publications [33–35]. The lentivirus-mediated shRNA for silencing HCN2 subunits (HCN2-shRNA) containing green fluorescence protein (GFP) and a non-targeting sequence as the negative control shRNA (Con-shRNA) were constructed by Genechem Corp., Ltd. (Shanghai, China). HCN2 expression in HT22 neurons was knocked down by lentivirus-mediated shRNA according to the manufacturer’s instructions. For lentivirus transduction, HT22 neurons at ~ 80% confluence were infected with lentivirus-bearing specific shRNAs in growth medium containing 8 µg/mL polybrene for 24 h, and then the infected neurons were subcultured for 48 h in growth medium. The transfection efficiency was further quantified by flow cytometric analysis. HCN2 immunofluorescence intensity and protein levels in HT22 neurons were measured 48 h after transduction.

Stereotaxic injection was performed as we described previously [36] under anesthesia with pentobarbital sodium (40 mg/kg, intraperitoneal injection, i.p.). Three weeks after shRNA infusion, we randomly selected half of the HCN2-shRNA-infected rats to determine the injection site and infection efficiency, and then carried out further studies as described below.

### Cell Viability Assay [37]

HT22 neurons were seeded into 96-well plates. 10 μL CCK8 (Dojindo, Kumamoto, Japan) detection solution was added to each well and incubated at 37 °C for 1 h. The optical density values were recorded at 450 nm with an ELISA reader (Tecan, Männedorf, Switzerland) and then the survival rate in each group was calculated.

### Flow Cytometric Analysis

HT22 neurons from each group were rinsed twice with phosphate-buffered saline (PBS), then re-suspended in 100 μL PBS (pH 7.4). The relative percentages of apoptotic and necrotic cells were calculated by fluorescence-activated cell sorting (FACS) analysis using Annexin V-PE/7-AAD or Annexin V-FITC/PI double staining. The final rate of apoptosis was measured on a BD FACSCantoII flow cytometer with BD FACSDiva software (BD Biosciences, San Jose, CA). These experiments were carried out following the manufacturer’s instructions.

### Establishment of the Transient Global Cerebral Ischemic Model

Adult male Sprague–Dawley rats of clean grade (approval number SCXK(E)2015-0018, No. 42000600032127), aged 2–3 months (weighing 220–250 g), were purchased from Hubei Provincial Laboratory Animal Public Service Center. The rats were given adaptive feeding (with a standard laboratory diet and water) for one week before experiments [30]. All experiments were approved by the Review Committee for the Care and Use of Laboratory Animals of Tongji Medical College, Huazhong University of Science and Technology. All efforts were made to minimize both the suffering and number of animals used. TGCI was induced *via* the 2-vessel occlusion model as described by Sun *et al*. [38]. Briefly, under anesthesia (pentobarbital sodium, 40 mg/kg, i.p.), blood was collected in a warmed heparinized syringe from the external jugular vein (2.5 mL/100 g), and then the bilateral common carotid arteries were temporarily occluded with small arterial clamps. After 20 min, the clamps were released and the extracted blood was slowly reinfused. Sham-operated animals received the same surgical procedures without blood extraction and occlusion. CQ was dissolved in sterile water and injected intracerebroventricularly (12.5 mg/kg) 2 h before TGCI through a 26-G needle at the following stereotaxic coordinates: 0.8 mm posterior to bregma, 1.5 mm lateral to the midline, and 3.6 mm ventral to the skull surface. Sterile saline was administered for vehicle control.

### Water Maze Task

Three days after TGCI injury, we began Morris water maze training as described previously [30, 36], and recorded swim speed, latency to escape onto the hidden platform, proportion of time spent in the target quadrant, and amount of time spent in the quadrant of the former platform position with a video camera linked to a computer-based image analyzer (Morris water-maze tracking system MT-200; Chengdu Technology and Market Co., Ltd, Chengdu, China).

### Western Blot Analysis

Total protein was extracted from cultured HT22 neurons and hippocampal CA1 tissue using RIPA lysis buffer (P0013B, Beyotime) as described in our reports [30, 39]. Protein concentration was determined using a BCA Protein Assay Kit (Pierce). For gel electrophoresis, total extracted protein from each sample (80 μg) was separated on 10% or 15% sodium dodecyl sulfate polyacrylamide gels and then transferred to polyvinylidene fluoride membranes (Millipore, Billerica, MA, USA), followed by blocking with 5% non-fat milk for 2 h at room temperature. Subsequently, the membranes were incubated with the primary antibodies anti-LC3 (1:1000, PM036, MBL), anti-mTOR (1:1000, 2983, Cell Signaling Technology), anti-phospho-mTOR (Ser^2448^) (1: 1000, 5536, Cell Signaling Technology), anti-ULK1 (1:1000, 8054, Cell Signaling Technology), anti-phospho-ULK1 (Ser^317^) (1:1000, 6887, Cell Signaling Technology), anti-phospho-ULK1 (Ser^757^) (1:1000, 6888, Cell Signaling Technology), anti-atg5 (1:500, NB110-53818, Novus), anti-p62 (1:1000, ab56416, Abcam), anti-beclin1 (1:1000, NB500-249, Novus), anti-HCN1 (1:800, NBP1-20250, Novus), anti-HCN2 (1:200, APC-030, Alomone Labs), anti-LAMP-1 (1:500, SC-20011, Santa Cruz Biotechnology), anti-cathepsin D (1:500, SC-377124, Santa Cruz Biotechnology), anti-neuronal nuclear antigen (NeuN) (1:2000, MAB377, Millipore), anti-GAPDH (1:5000, cw0100, Cwbiotech), or anti-alpha tubulin (1:5000, ab125267, Abcam). The antigen-antibody complexes were visualized with goat anti-rabbit or goat anti-mouse horseradish peroxidase (HRP)-conjugated secondary antibodies (1:5000; Proteintech Group Inc., China) by using Immobilon Western chemiluminescent HRP substrate (WBKLS0500, Millipore). The optical density of bands was measured using NIH ImageJ software, and results were normalized to GAPDH or alpha tubulin in each sample lane. All assays were performed at least three times.

### Immunofluorescence and Hematoxylin & Eosin (H&E) Staining

HT22 neurons and rat brain sections were prepared for immunofluorescence as previously described [30, 40]. Subsequently, HT22 neurons were incubated with the primary antibody against anti-LC3 and/or anti-LAMP-1, anti-HCN1, or anti-HCN2 for 2 h at 37 °C, then with DyLight 488 Affinipure goat anti-rabbit IgG (H+L) (A23220, Abbkine, CA), DyLight 549 Affinipure goat anti-mouse IgG (H+L) (A23310, Abbkine), or DyLight 549 Affinipure rabbit anti-sheep IgG (H+L) (313-505-003, Jackson) for 2 h at 37 °C. Immunohistochemical staining of sections was sequentially performed following incubation with anti-LC3 (1: 500, PM036, MBL) overnight at 4 °C and Fluorescein (FITC)-conjugated Affinipure donkey anti-rabbit IgG(H+L) (SA00003-8, Proteintech Group Inc., China) for 2 h. And the images were recorded and analyzed using an Olympus FluoView 1200 confocal microscope system (Olympus Corp., Tokyo, Japan). LC3-postitive puncta and LAMP-1-positive lysosomes were then analyzed using ImageJ. LC3-positive puncta were expressed as a percentage of the total puncta within the indicated size ranges or co-localized with LAMP-1-positive lysosomes. The fluorescence intensity of LC3 staining in the rat hippocampal CA1 area was estimated using ImageJ. H&E staining was performed according to a protocol described previously [30] and photographed under the microscope. The number of neurons was counted using ImageJ and neuronal density was calculated as the ratio of viable neuron counts to the area of view in a section.

### Statistical Analysis

All data are presented as the mean ± SD. Statistical analyses were calculated by one- or two-way analysis of variance (ANOVA, Tukey’s HSD [honestly significant difference] test) using SPSS 22 .0 software (SPSS Inc., USA). Differences between two groups were evaluated by the *t*-test. *P *< 0.05 was considered statistically significant.

## Results

### ZD7288 Protects HT22 Neurons Against OGD/R Injury

**Fig. 1:**
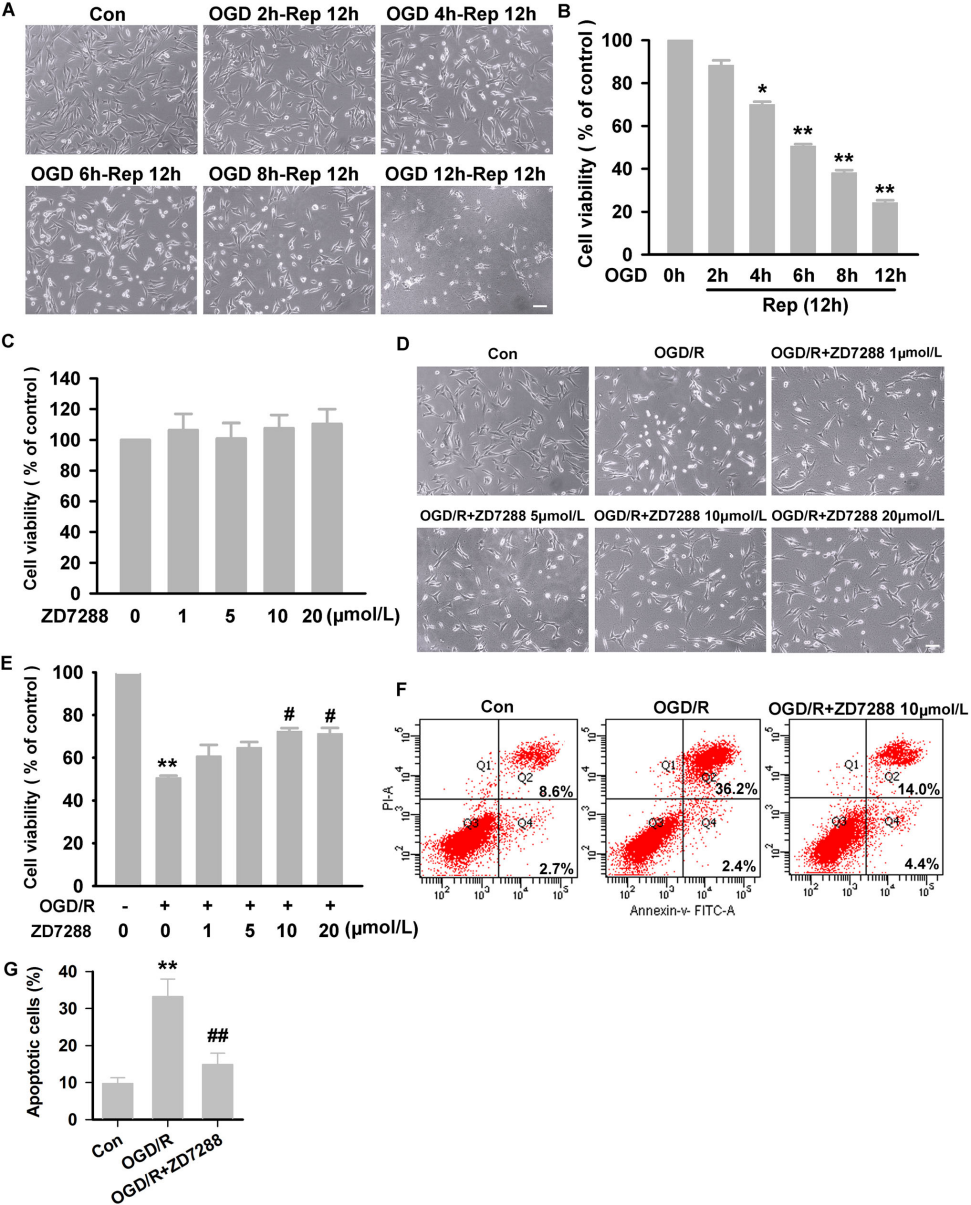
Neuroprotection against OGD/R injury by ZD7288. **A** Representative light microscopic live-cell images of HT22 neurons exposed to OGD insults of different durations (2 h, 4 h, 6 h, 8 h, or 12 h) followed by a further 12 h of reperfusion (× 200, scale bar, 200 μm). **B** Cell viability after OGD insults of the different durations followed by reperfusion (CCK8 assay). **C** ZD7288 at all concentrations used (1 μmol/L, 5 μmol/L, 10 μmol/L, and 20 μmol/L) did not affect the viability of normoxic neurons. **D** Representative light microscopy of HT22 neurons pretreated with ZD7288 at the different concentrations. **E** Effect of different concentrations of ZD7288 on HT22 neuronal viability after OGD/R (using CCK8). **F** Representative dot plots of flow cytometric analysis of cell death in HT22 neurons. **G** Quantitative analysis of the apoptotic rates by flow cytometry. Experiments were performed at least three times with similar results. *P < 0.05 and **P < 0.01 *versus* control group; ^#^*P* < 0.05 and ^##^*P* < 0.01 *versus* OGD/R group.

Compared with the control group, the degree of neuronal damage was gradually aggravated after exposure to OGD insults of different durations (2 h, 4 h, 6 h, 8 h, or 12 h) followed by a further 12 h of reperfusion; the viability of HT22 neurons decreased from 100% to 88.28% ± 2.29%, 70.00% ± 1.38%, 50.50% ± 1.02%, 38.07% ± 1.28%, and 24.16% ± 1.25%, respectively (Fig. 1A, B). And the appropriate model was determined to be 6-h OGD followed by 12-h reperfusion because this caused nearly half of the neurons to die.

To determine whether ZD7288 protects HT22 neurons from damage induced by OGD/R, the neurons were pretreated with ZD7288 at different concentrations (1 μmol/L, 5 μmol/L, 10 μmol/L and 20 μmol/L) 2 h before OGD, and cell viability was measured at the end of OGD/R. At all these concentrations, ZD7288 had no effect on the viability of normoxic neurons as assayed by CCK-8 (Fig. 1C). Compared to OGD/R alone (50.53% ± 1.02%), the neuronal viability increased with ZD7288 concentration (1 μmol/L, 5 μmol/L, and 10 μmol/L) (60.69% ± 5.29%, 64.63% ± 2.71%, and 72.27% ± 1.58%); ZD7288 at 20 μmol/L did not further increase the viability (71.19% ± 2.74%) (Fig. 1D, E). Thus, 10 μmol/L ZD7288 was used in the subsequent experiments. Next, necrosis and/or apoptosis of neurons was analyzed by flow cytometry. After OGD/R, HT22 neurons were stained with propidium iodide (PI) and FITC-labeled Annexin V (AV-FITC). Our results showed that OGD/R resulted in a significant increase in the percentage of apoptotic neurons (AV^+^/PI^−^ and AV^+^/PI^+^) from a control value of 11.3% to 38.6%. ZD7288 (10 μmol/L) remarkably decreased the percentage of apoptotic neurons (18.4%) induced by OGD/R (Fig. 1F, G).

### ZD7288 Corrects the Excessive Autophagy Induced by OGD/R Injury in HT22 Neurons

**Fig. 2:**
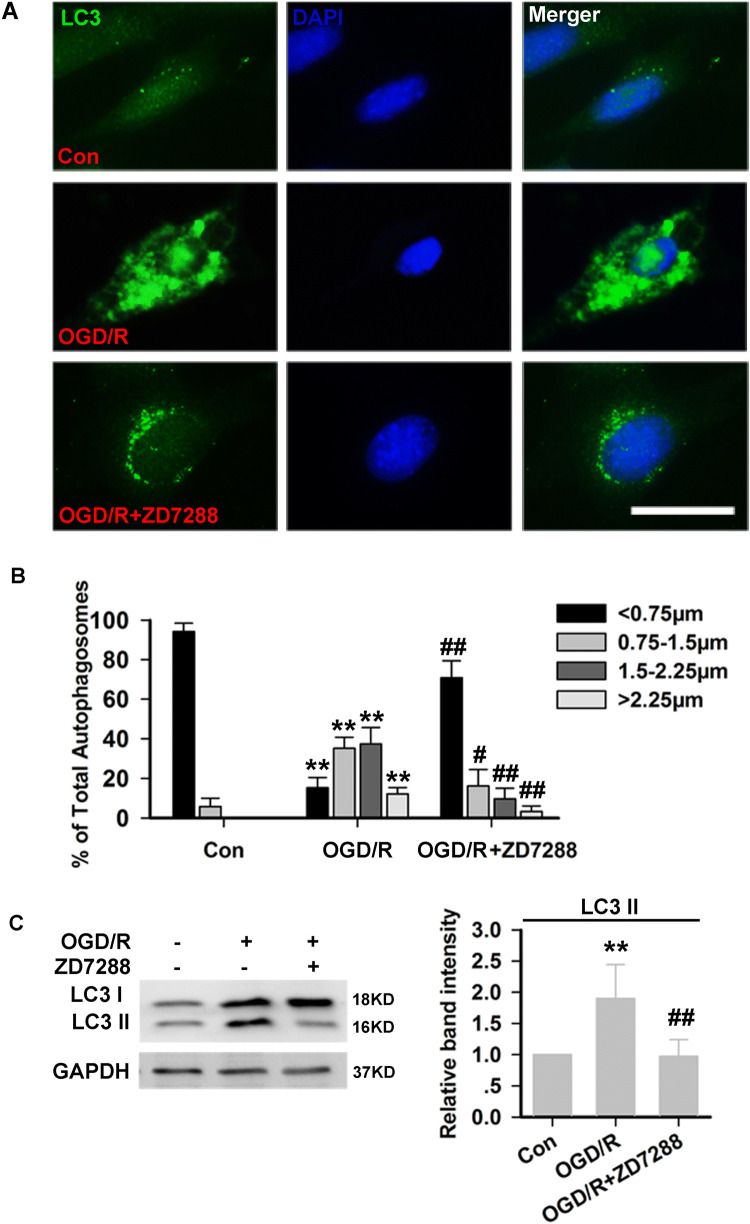
ZD7288 corrects the excessive autophagy induced by OGD/R injury in HT22 neurons. **A** Representative photomicrographs of immunohistochemical staining with anti-LC3 antibody in HT22 neurons (scale bar, 50 μm). **B** Size distributions of autophagosome vacuoles (percentages of diameters within the indicated ranges of all LC3-positive puncta per neuron). **C** Expression of LC3-II protein in HT22 neurons. Experiments were performed at least four times with similar results. **P < 0.01 *versus* control group; ^#^*P* < 0.05, ^##^*P* < 0.01 *versus* OGD/R group.

Compared with the control group, LC3 immunoreactivity was robustly elevated in the OGD/R group, whereas in the ZD7288 (10 μmol/L)+OGD/R group, the LC3 immunoreactivity declined towards basal levels (Fig. 2A, B). To further confirm that ZD7288 corrected OGD/R-induced autophagy, LC3-II (a marker of autophagosomes) was detected by Western blot analysis. Our results showed that the LC3-II levels were significantly increased in the OGD/R group. However, in the ZD7288 (10 μmol/L)+OGD/R group, the levels of LC3-II were remarkably decreased compared with the untreated OGD/R group (Fig. 2C).

### Influence of ZD7288 on the Regulation of Autophagosome Formation, Lysosomal Enzymes, and Numbers of Lysosomes

**Fig. 3:**
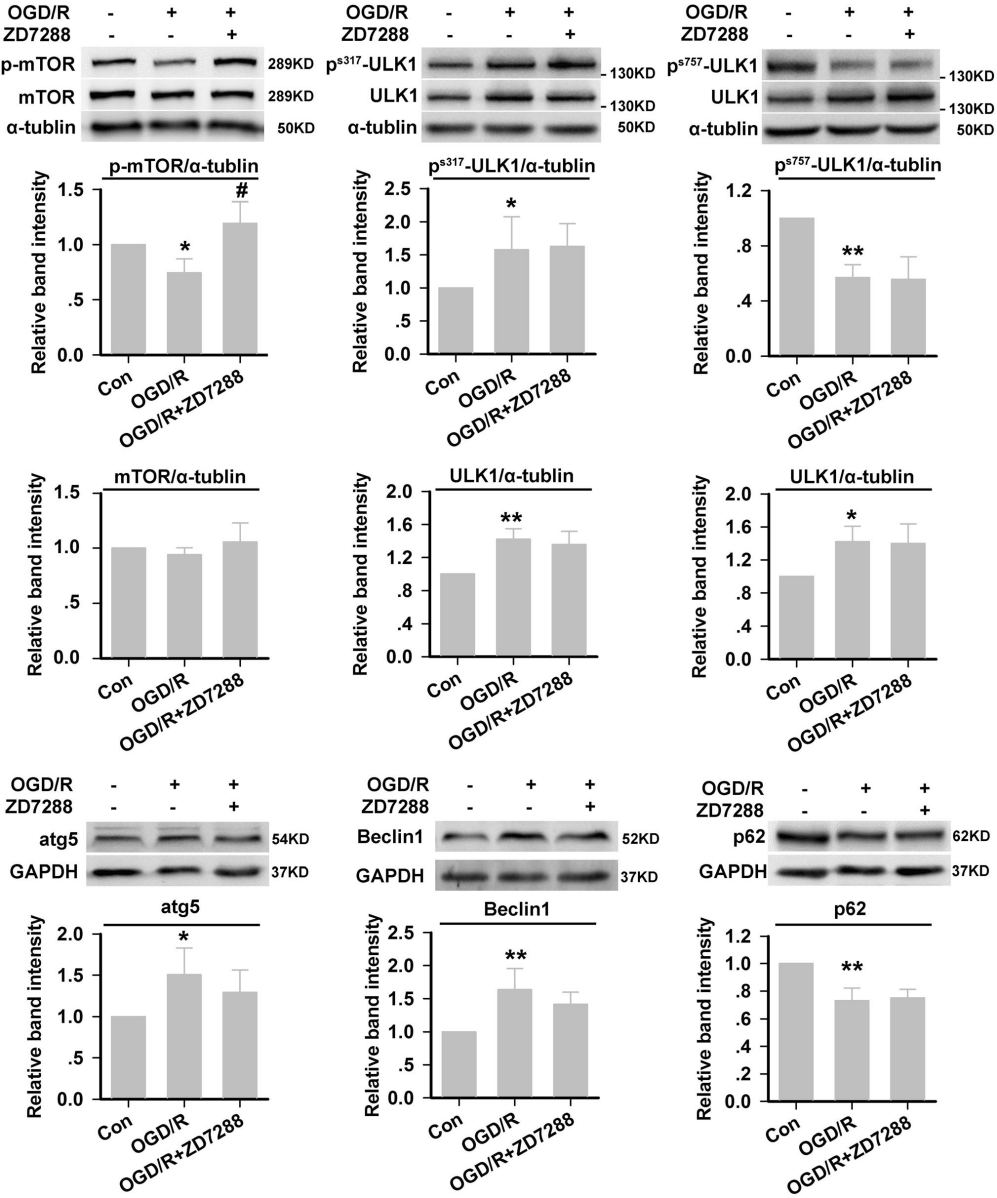
The expression of autophagic proteins in HT22 neurons. The experiments were performed at least four times with similar results. *P < 0.05 and **P < 0.01 *vs* control group; ^#^*P* < 0.05 *versus* OGD/R group.

**Fig. 4:**
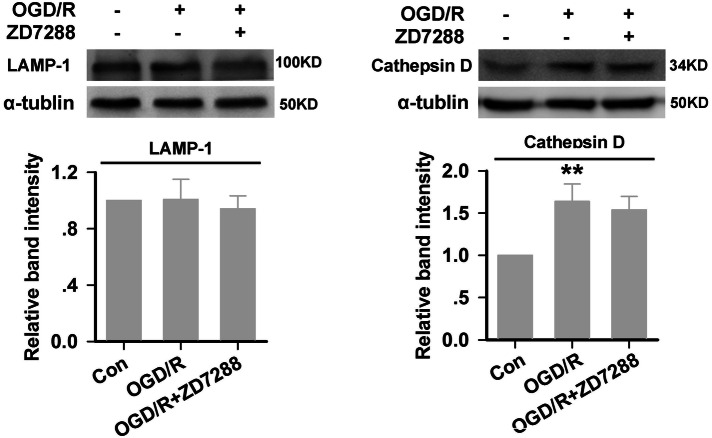
The expression of LAMP-1 and cathepsin D in HT22 neurons. Experiments were performed at least four times with similar results. **P < 0.01 *versus* control group.

To determine how ZD7288 regulates autophagy, we first analyzed the expression of regulators of autophagosome formation. Our results showed that, in the OGD/R group, p-mTOR, p-ULK1 (Ser^757^), and p62 were significantly decreased, while p-ULK1 (Ser^317^), Beclin1, and atg5 were significantly increased. ZD7288 (10 μmol/L) reversed the change in p-mTOR expression, but did not change the expression of p-ULK1 (Ser^757^), p-ULK1 (Ser^317^), p62, beclin1, or atg5 compared with OGD/R alone (Fig. 3). Next, we investigated the influence of ZD7288 on lysosomes and lysosomal enzymes, and found that OGD/R induced a significant increase in cathepsin D expression, but not LAMP-1. Treatment of the OGD/R group with ZD7288 at 10 μmol/L did not change the expression of cathepsin D or LAMP-1 (Fig. 4).

### ZD7288 Accelerates Autophagic Degradation in HT22 Neurons During OGD/R Injury

**Fig. 5:**
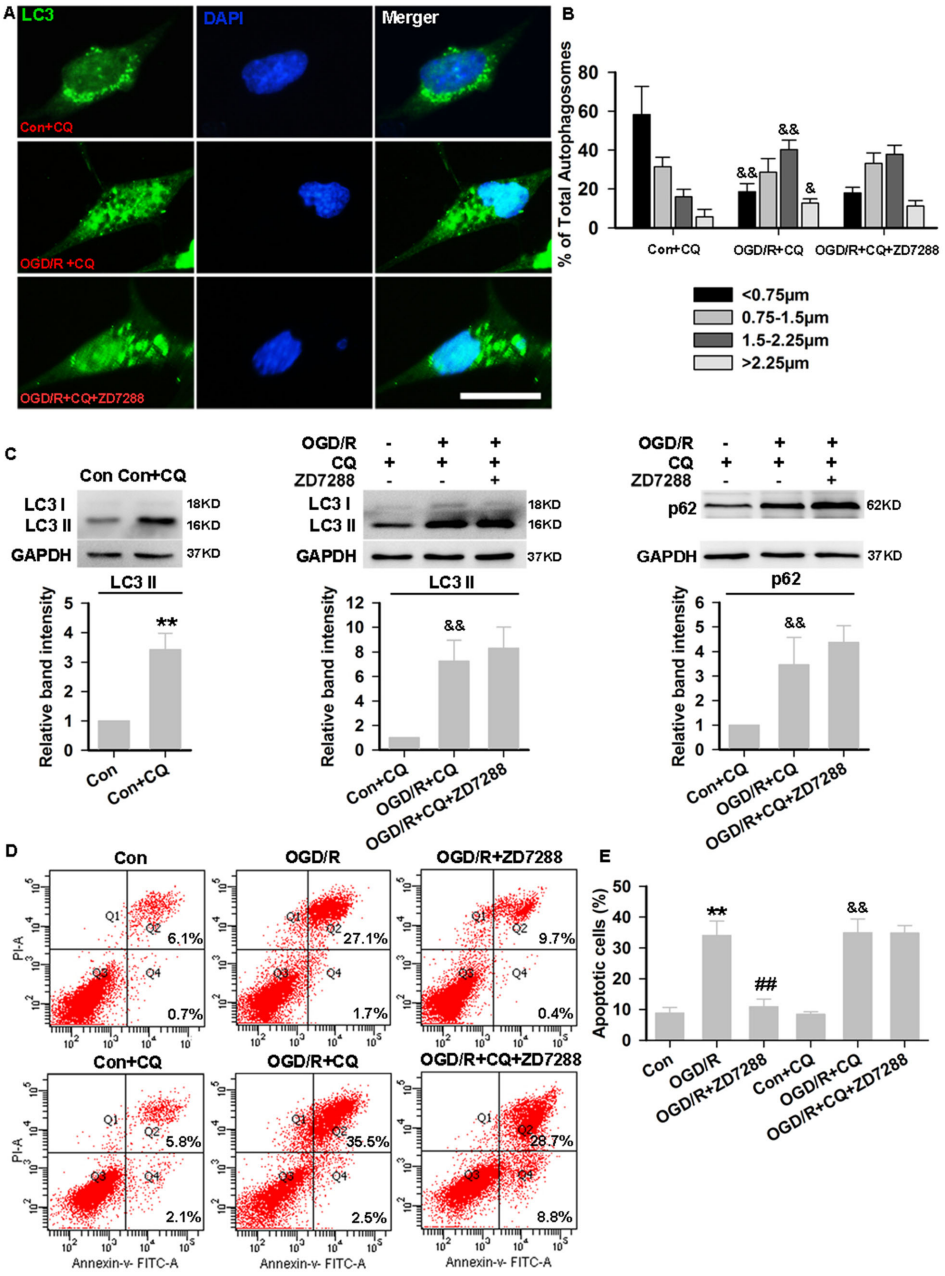
ZD7288 accelerates autophagic degradation in HT22 neurons during OGD/R injury. **A** Representative photomicrographs of immunohistochemical staining with anti-LC3 antibody in HT22 neurons (scale bar, 50 μm). **B** Size distributions of autophagosome vacuoles (percentages of diameters within the indicated ranges of all LC3-positive puncta per neuron). **C** Protein expression of LC3-II and p62 in HT22 neurons. **D** Representative dot plots of flow cytometric analysis of cell death in HT22 neurons. **E** Quantitative analysis of the apoptosis rates by flow cytometry. The experiments were performed at least three times with similar results. **P < 0.01 *versus* control group, ^##^*P* < 0.01 *versus* OGD/R group, ^&^*P* < 0.05, ^&&^*P* < 0.01 *versus* Con+CQ group.

To determine whether the correction of excessive autophagy by ZD7288 is due to the promotion of autophagic degradation, we further examined changes in autophagy-related proteins after co-incubation with CQ (50 μmol/L), which inhibits autophagosome–lysosome fusion. First, LC3 immunostaining was assessed in the presence of CQ with or without ZD7288. CQ increased the LC3 puncta in the control and OGD/R groups, and ZD7288 failed to correct the excessive autophagy in the presence of CQ (Fig. 5A, B). Consistent with the immunocytochemical data, the addition of CQ increased the levels of LC3-II in controls, representing the maximal autophagic flux. In the OGD/R group, CQ increased the LC3-II levels to a greater extent. However, in the presence of CQ, treatment with ZD7288 at 10 μmol/L in the OGD/R group did not change the excessive expression of LC3-II and p62 (Fig. 5C). In order to further explore whether neuroprotection against OGD/R injury by ZD7288 is due to the acceleration of autophagic degradation, we measured the numbers of vital, apoptotic, and necrotic neurons in the presence of CQ by flow cytometry. CQ at 50 μmol/L had no significant effect on apoptosis or necrosis in HT22 neurons. However, it eliminated the neuroprotection by ZD7288 against OGD/R injury (Fig. 5D, E).

### Neuroprotection Against OGD/R Injury by Blocking HCN2 Channels Using Genetic Knockdown is Due to Promotion of Autophagic Degradation in HT22 Neurons

**Fig. 6:**
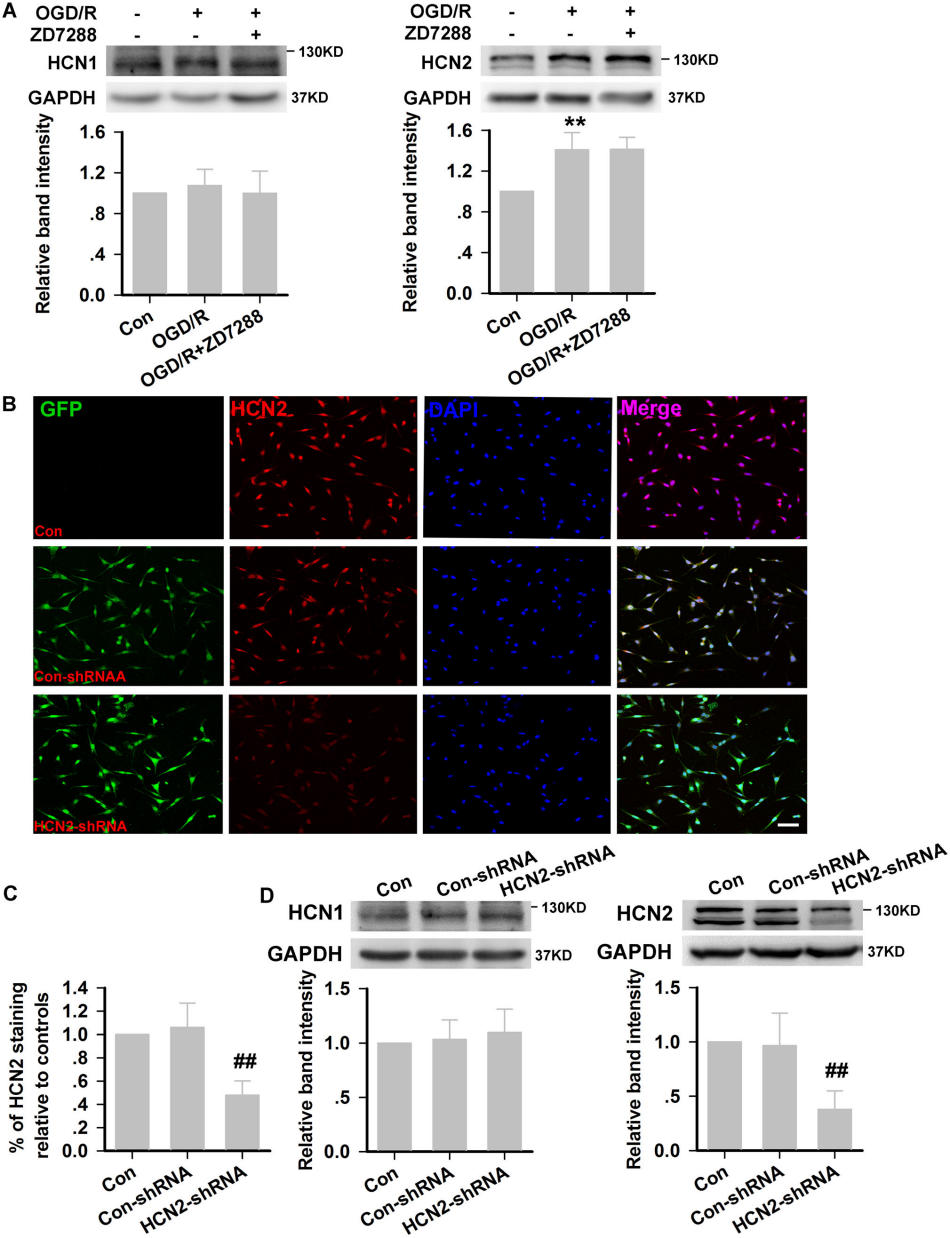
HCN2 subunit knockdown in HT22 neurons. **A** HCN1 and HCN2 expression in HT22 neurons after OGD/R injury (experiments were performed at least four times with similar results). **B** Representative photomicrographs of fluorescence produced by EGFP and immunohistochemical staining with anti-HCN2 antibody in HT22 neurons (× 200, scale bar, 200 μm). **C** Quantitative analysis of HCN2 immunoreactivity (experiments were performed at least three times with similar results). **D** Relative expression of HCN1 and HCN2 channels at the protein level in Con-shRNA and HCN2-shRNA cells (experiments were performed at least four times with similar results) **P < 0.01 *versus* control group; ^##^*P *< 0.01 *versus* Con-shRNA group.

At the end of OGD/R injury, HCN1 channels remained unchanged, while HCN2 channels were significantly increased in HT22 neurons (Fig. 6A). To further confirm that ZD7288 accelerated the autophagic degradation by blocking HCN channels, we next blocked HCN2 channels by genetic knockdown, and assessed the expression of EGFP in the lentivirus using confocal laser scanning microscopy. As shown in Fig. 5B, the cell morphology in each group did not differ significantly. EGFP was strongly expressed in HT22 neurons transfected with the lentivirus. The percentage of EGFP-positive cells exceeded 90% in the HCN2- or empty vector-transfected (HCN2-shRNA or Con-shRNA) HT22 neurons, as determined by flow cytometry (data not shown). HCN2-shRNA significantly reduced the fluorescence intensity of HCN2 (Fig. 6B, C) but not HCN1 (data not shown). Consistent with the immunocytochemistry, the relative expression of HCN2 channels at the protein level was inhibited by 63.39% ± 3.46% in HCN2-shRNA cells relative to Con-shRNA cells, and there was no effect on HCN1 channel expression (Fig. 6D).

**Fig. 7:**
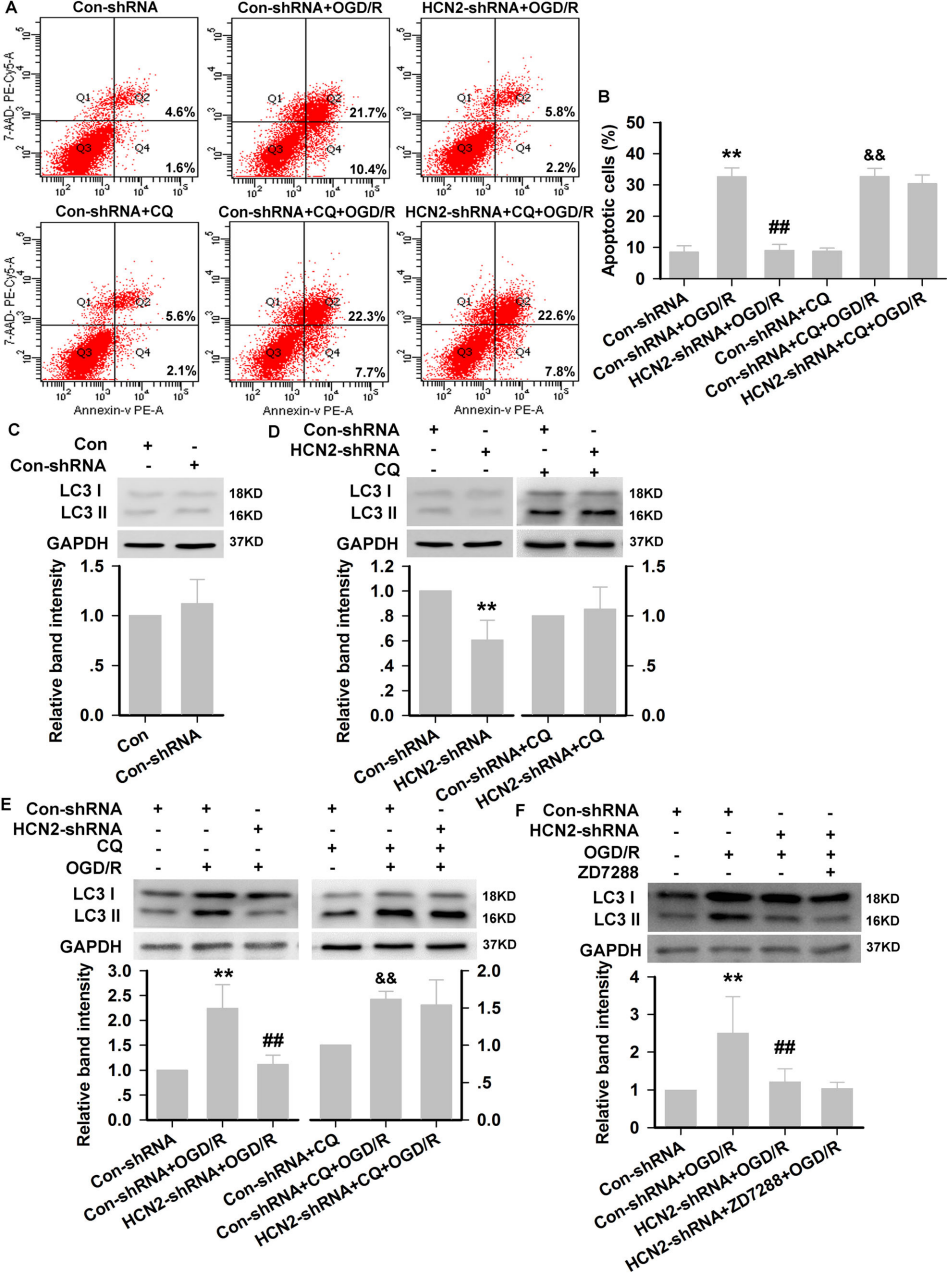
Neuroprotection against OGD/R injury by blocking HCN2 channels using genetic knockdown is due to promotion of autophagic degradation. **A** Representative dot plots of flow cytometry analysis of cell death in HT22 neurons. **B** Quantitative analysis of the apoptosis rates by flow cytometry (experiments were performed at least three times with similar results). **C–F** Protein expression of LC3-II in each group (experiments were performed at least four times with similar results). **P < 0.01 *versus* Con-shRNA group; ^##^*P* < 0.01 *versus* Con-shRNA+OGD/R group; ^&&^*P* < 0.01 *versus* Con-shRNA+CQ group.

Our results showed that Con-shRNA had no significant effect on apoptosis in HT22 neurons. OGD/R resulted in a significant increase in the percentage of apoptotic neurons from a control value of 6.2% to 32.1% in Con-shRNA-transfected HT22 neurons. The percentage of apoptotic neurons induced by OGD/R remarkably decreased in HCN2-shRNA-transfected neurons (8.0%). However, CQ (50 μmol/L) eliminated the neuroprotection by HCN2 channel knockdown (Fig. 7A, B). Next, we evaluated the effect of HCN2 channel knockdown on autophagic flux. A slight increase in LC3-II levels occurred in Con-shRNA-transfected neurons, but the difference from normal neurons was not significant (Fig. 7C). Under normal culture conditions, HCN2-shRNA decreased the LC3-II level, and CQ significantly increased the LC3-II level to a great extent. Besides, in the presence of CQ, HCN2-shRNA did not reverse the excessive expression of LC3-II (Fig. 7D). In Con-shRNA-transfected HT22 neurons, western blot analysis revealed that exposure to OGD/R resulted in an increase in the LC3-II level, while knockdown of HCN2 channels corrected the excessive expression of LC3-II. However, in the presence of CQ, knockdown of HCN2 channels did not reverse the excessive expression of LC3-II induced by OGD/R (Fig. 7E). In HCN2-shRNA-transfected neurons, ZD7288 (10 μmol/L) decreased the LC3-II level to a lesser extent at the end of OGD/R, and did not significantly differ from that of HCN2-shRNA transfection alone (Fig. 7F).

### HCN2-shRNA Promotes Autophagosome–Lysosome Fusion in HT22 Neurons

**Fig. 8:**
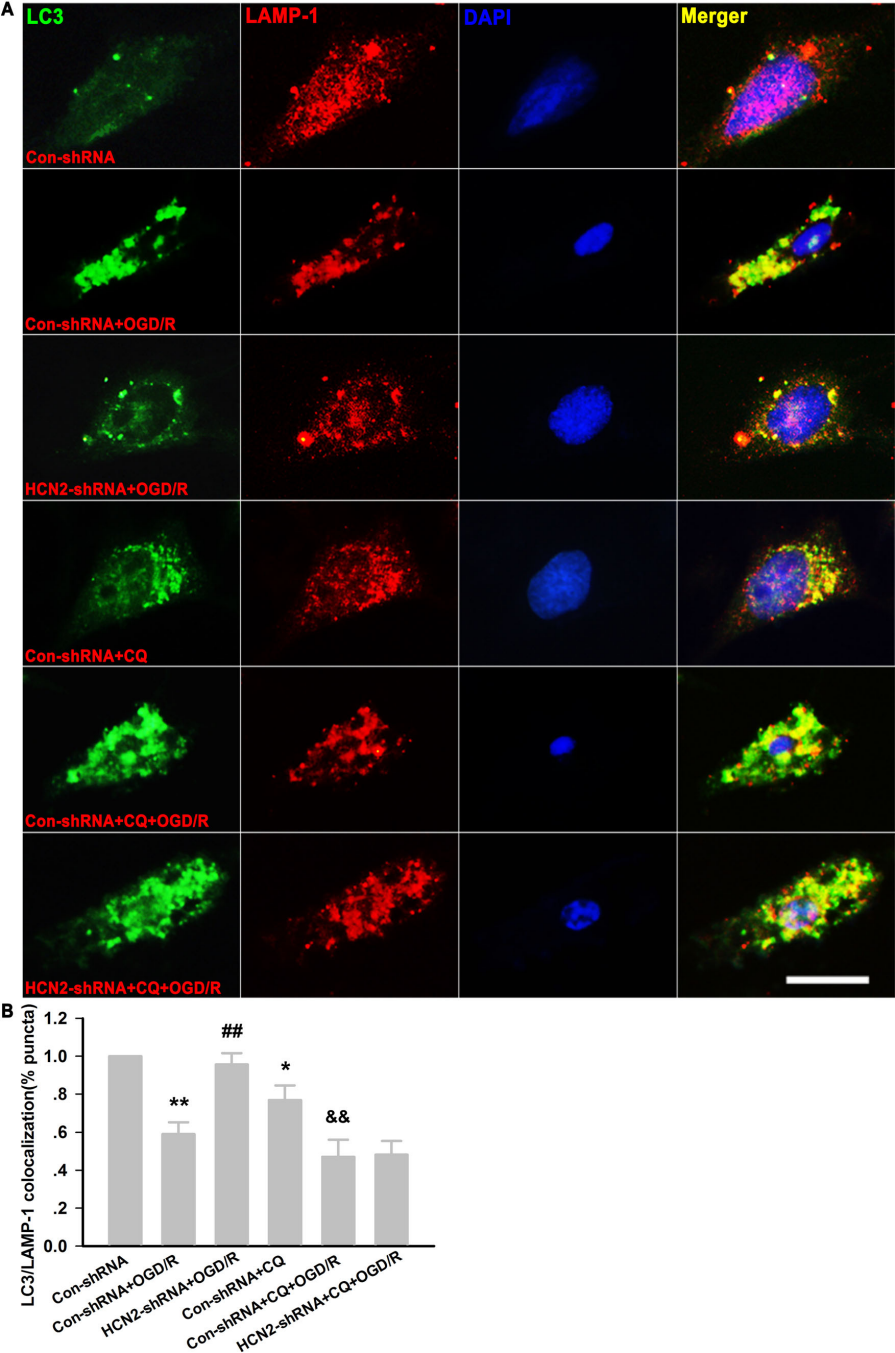
HCN2-shRNA promotes autophagosome-lysosome fusion in HT22 neurons. **A** Representative images of immunohistochemical staining with anti-LC3 and anti-LAMP-1 in HT22 neurons (scale bar, 50 μm). **B** Analysis of the percentages of LC3-positive puncta that co-localize with LAMP-1-positive lysosomes in each group. *P < 0.05, **P < 0.01 *versus* Con-shRNA group, ^##^*P* < 0.01 *versus* Con-shRNA+OGD/R group, ^&&^*P* < 0.01 *versus* Con-shRNA+CQ group.

In order to further evaluate the effect of HCN2-shRNA on the fusion of autophagosomes and lysosomes, we used immunohistochemical co-staining with antibodies against LC3 and LAMP-1 in HT22 neurons. As shown in Fig. 8, consistent with the western blot results, exposure of Con-shRNA-transfected neurons to OGD/R resulted in an increase in LC3 puncta, while knockdown of HCN2 channels significantly reduced them. However, in the presence of CQ, knockdown of HCN2 channels did not reverse the excessive autophagy induced by OGD/R. Besides, the expression of LAMP-1 did not change significantly in each group. Furthermore, we found that the percentage of LC3-positive puncta co-localized with LAMP-1-positive lysosomes decreased in Con-shRNA-transfected neurons exposed to OGD/R or CQ. In HCN2-shRNA-transfected HT22 neurons, the percentage of LC3-positive puncta that co-localized with LAMP-1-positive lysosomes increased under OGD/R; however, this percentage was significantly decreased by addition of CQ to the HCN2-shRNA-transfected neurons. Our results suggested that HCN2-shRNA promotes the fusion of autophagosomes and lysosomes in HT22 neurons.

### Blocking HCN2 Channels by Genetic Knockdown Protects Against TGCI in Rats

**Fig. 9:**
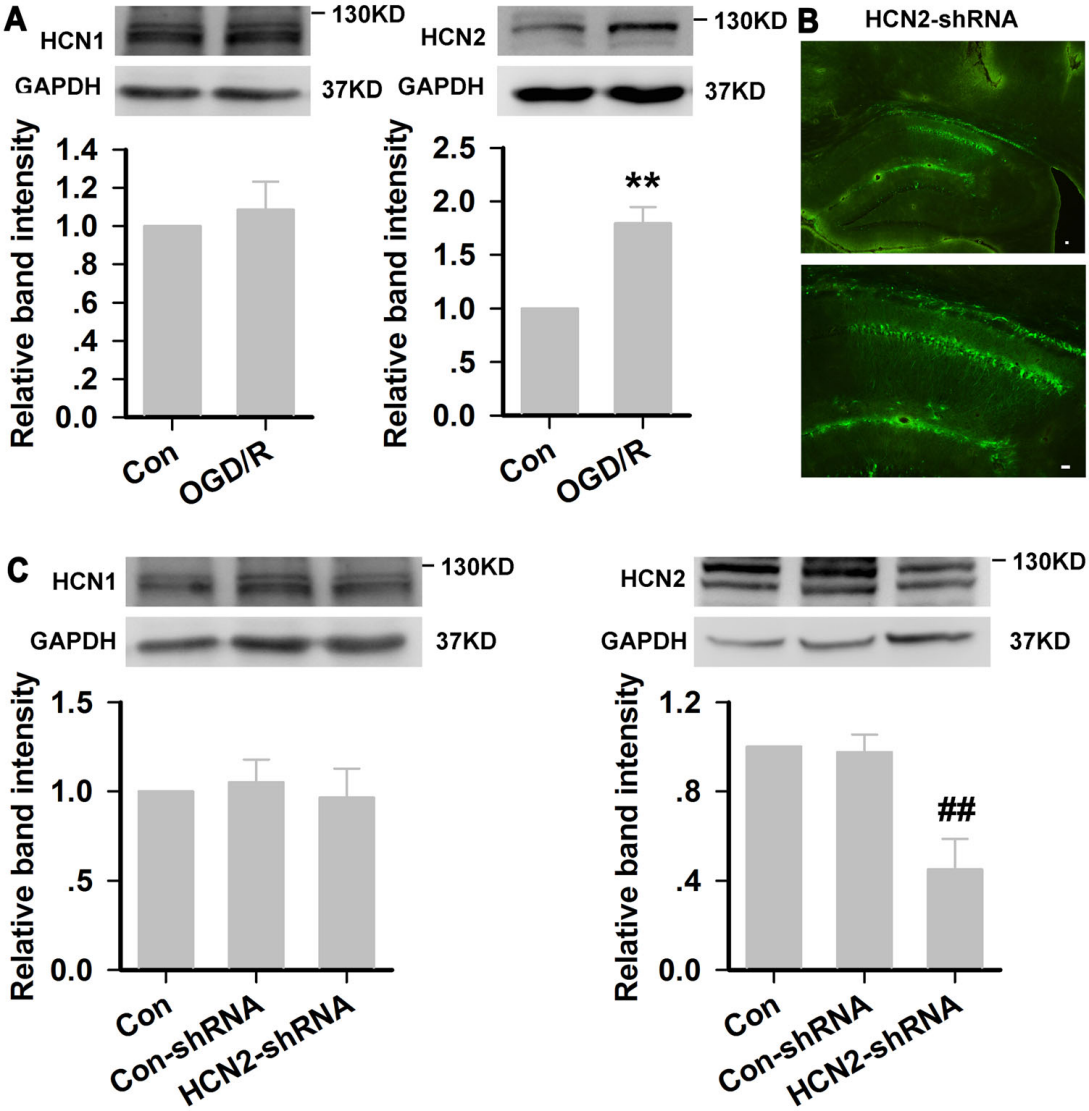
HCN2 subunit knockdown in rat hippocampal CA1 region. **A** HCN1 and HCN2 expression in CA1 after TGCI (experiments were performed at least four times with similar results). **B** Distribution of lentivirus in the dorsal hippocampal CA1 region (green; × 40, × 100, scale bars, 100 μm). **C** Relative expression of HCN1 and HCN2 channels at the protein level in Con-shRNA and HCN2-shRNA rats (experiments were performed at least four times with similar results). **P < 0.01 *versus* Con group; ^##^*P *< 0.01 *versus* Con-shRNA group.

After TGCI, HCN1 channels remained unchanged, however, HCN2 channels were significantly increased in the rat hippocampal CA1 area (Fig. 9A). To further evaluate the potential protective effects against TGCI, we next blocked HCN2 channels by genetic knockdown. The relative expression of HCN2 channels at the protein level was inhibited by 54.92% ± 1.37% in CA1 compared with Con-shRNA rats, and it had no effect on HCN1 channel expression (Fig. 9C).

**Fig. 10:**
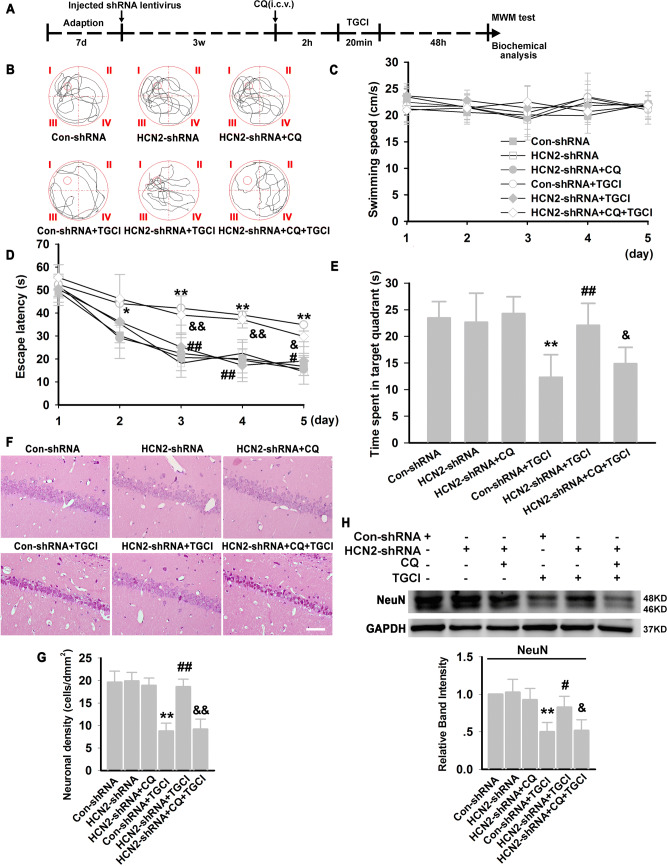
Neuroprotection against TGCI by blocking HCN2 channels with genetic knockdown. **A** Experimental design. **B** Typical swimming paths of each group. **C** Average speed from day 1 to day 5 (*n = *8 per group). **D** Escape latency to find the hidden platform from day 1 to day 5. **E** Percentage of quadrant dwell time in the target quadrant on training day 6. **F** Example of H&E-stained sections of the hippocampus in each group (scale bar, 100 μm). **G** Quantification of neuronal density in CA1. **H** Protein expression of NeuN in CA1 of each group (*n = *4 per group). *P < 0.05, **P < 0.01 *versus* Con-shRNA group; ^#^*P* < 0.05, ^##^*P* < 0.01 *versus* Con-shRNA+TGCI group; ^&^*P* < 0.05, ^&&^*P* < 0.01 *versus* HCN2-shRNA+TGCI group. w, weeks.

Then, we examined the effect of blocking HCN2 channels on performance in the Morris water maze task and found no significant difference in swimming speed between groups (Fig. 10C). The latency to find the platform of TGCI rats was longer than the rats in the Con-shRNA group (Fig. 10D). The HCN2-shRNA+TGCI group showed a learning latency similar to that of the Con-shRNA group. However, in the presence of CQ, knockdown of HCN2 channels did not reverse the prolongation of latency to find the platform of TGCI rats (Fig. 10E). Furthermore, the rats in the Con-shRNA group spent longer in the target quadrant than TGCI rats (Con-shRNA group: 23.46% ± 3.08%, TGCI rats: 12.32% ± 4.22%). HCN2-shRNA improved the TGCI-induced deficit of acquisition in the water maze (HCN2-shRNA+TGCI group: 22.08% ± 4.12%), and CQ (12.5 mg/kg) eliminated the effect of HCN2-shRNA (HCN2-shRNA+CQ+TGCI group: 14.87% ± 3.07%, Fig. 10E).

In the present study, H&E staining and Western blotting were used to assess the influence of HCN2-shRNA on degenerative changes in the hippocampal CA1 area. Forty-eight hours after TGCI, significant neuronal loss in CA1 was detected on H&E staining (Fig. 10F, G), and HCN2-shRNA markedly diminished this neuronal loss. Consistent with the above results, Western blotting showed that HCN2-shRNA reversed the TGCI-induced reduction of NeuN protein in CA1. However, in the presence of CQ, knockdown of HCN2 channels did not reverse the TGCI-induced neuronal loss in CA1 (Fig. 10H).

### HCN2-shRNA Corrects Excessive Autophagy Induced by TGCI in Rats

**Fig. 11:**
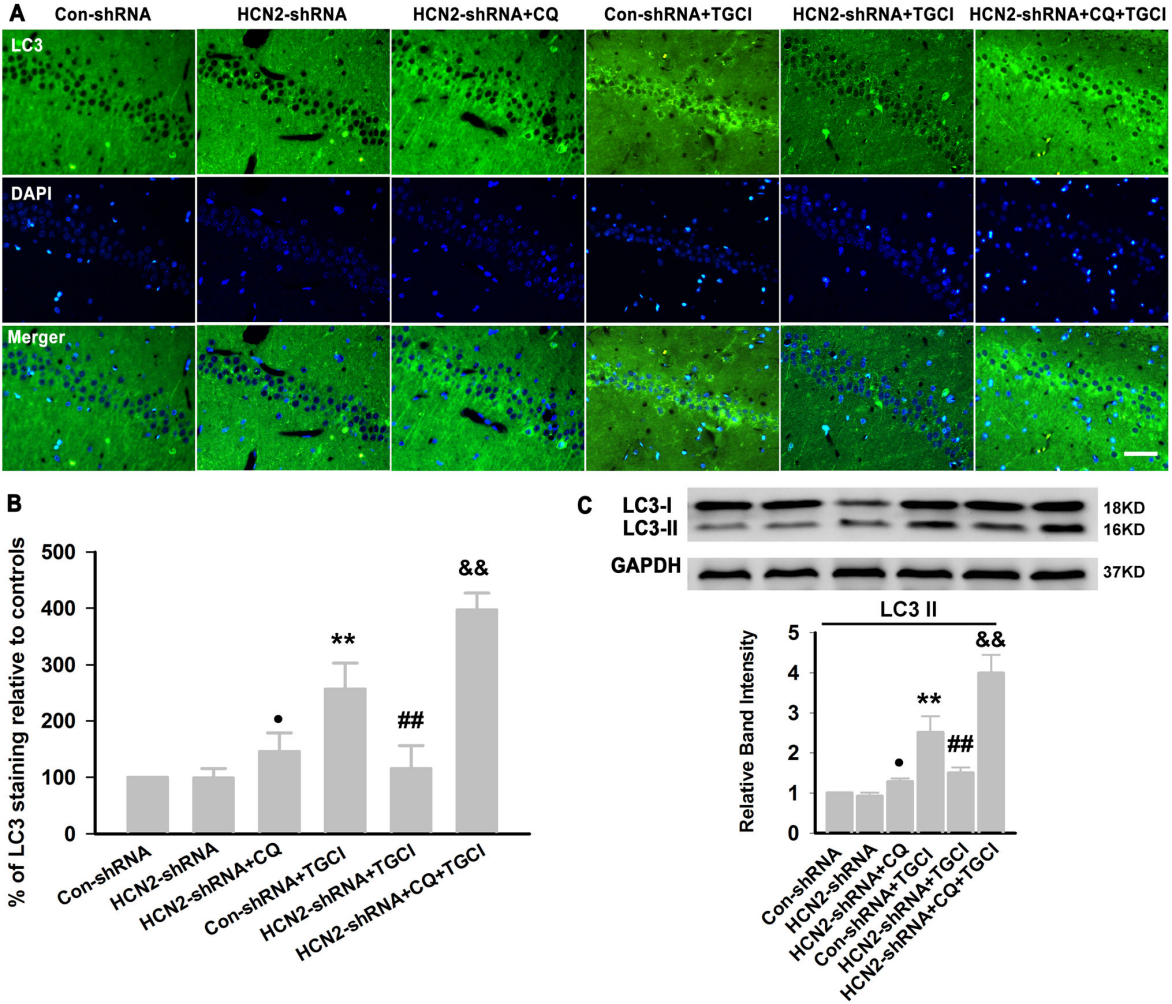
HCN2-shRNA corrects the excessive autophagy induced by TGCI in rats. **A** Representative photomicrographs of immunohistochemical staining with anti-LC3 antibody in the hippocampal CA1 area (scale bar, 100 μm). **B** Quantitative analysis of the LC3 immunoreactivity (*n = *4 per group). **C** Protein expression of LC3-II in CA1 of each group (*n = *4 per group). ^●^*P* < 0.05 *versus* HCN2-shRNA group, **P < 0.01 *versus* Con-shRNA group; ^##^*P* < 0.01 *versus* Con-shRNA+TGCI group; ^&&^*P* < 0.01 *versus* HCN2-shRNA+TGCI group.

To investigate whether autophagy is involved in the neuroprotection by blocking HCN2 channels under TGCI, we examined the activation of autophagy in the hippocampal CA1 area. Forty-eight hours after TGCI, the LC3 immunoreactivity was significantly increased in CA1 (Fig. 11A, B), consistent with the protein expression of LC3-II (Fig. 11C). Knockdown of HCN2 channels corrected the excessive expression of LC3-II. However, in the presence of CQ, knockdown of HCN2 channels did not reverse the excessive expression of LC3-II induced by TGCI (Fig. 11A–C).

## Discussion

In the present study, we demonstrated for the first time that blocking HCN channels by genetic knockdown or pharmacology protects hippocampal neurons from the damage induced by OGD/R and TGCI, and this might be attributed to the accelerated autophagic degradation.

Our results showed that, compared to untreated OGD/R, pretreatment with ZD7288 (10 μmol/L) significantly increased neuronal viability as assayed by CCK-8. However, OGD/R not only resulted in neuronal necrosis and/or apoptosis, but also decreased the HT22 cell proliferation rate. So we further analyzed the necrosis and apoptosis of neurons by flow cytometry. Our results showed that ZD7288 (10 μmol/L) also remarkably decreased the percentage of apoptotic neurons induced by OGD/R. The present results suggested that ZD7288 has protective effects against OGD/R-induced injury in hippocampal HT22 neurons.

Many reports have shown that the excessive or uncontrolled autophagy induced by ischemic injury leads to autophagic cell death, which is a form of non-apoptotic programmed cell death characterized by the presence of intense autophagy [41–43], and the correction of excessive autophagy can attenuate cerebral ischemia-associated neuronal damage [14–17]. Consistent with these studies, we found that ZD7288 corrected the excessive autophagy induced by OGD/R injury in HT22 neurons. To determine how ZD7288 regulates autophagy, we first analyzed the expression of regulators of autophagosome formation. Our results showed that ZD7288 (10 μmol/L) remarkably increased the phosphorylation of mTOR (Ser^2448^) under OGD/R conditions in HT22 neurons. However, ZD7288 (10 μmol/L) did not reverse the OGD/R-induced decrease in phosphorylation of ULK1 at Ser^757^. Previous reports have shown that high mTOR activity prevents ULK1 activation by phosphorylating ULK1 Ser^757^ [44] and the subsequent inhibition of autophagy [45]. We hypothesized that upregulation of p-mTOR (Ser^2448^) might act as a rescue mechanism to counter ZD7288-mediated effects in the HT22 neurons. A decrease in the phosphorylation of ULK-1 at Ser^757^ promotes its release from mTOR and association with AMPK, which enables the phosphorylation of ULK-1 at Ser^317^ by AMPK, and then activates beclin1, accelerating LC3-I transformation into an active lapidated form (LC3-II) [46]. Therefore, we further analyzed the expression of phosphorylation of ULk1 at Ser^317^, beclin1, and atg5, and found that OGD/R increased the expression of all three, and treatment with ZD7288 at 10 μmol/L did not change their expression compared with the OGD/R group. These results are consistent with our hypothesis that p-mTOR levels are upregulated as a feedback mechanism to counter ZD7288-mediated effects in HT22 neurons, which means that ZD7288 is not involved in the regulation of autophagosome formation through activation of p-mTOR and p-AMPK.

Studies have shown that brain ischemia causes a late-stage block of autophagy, which prevents the autophagic degradation during autophagic flux [47], and leads to the accumulation of intracellular protein aggregates and damaged organelles, and delayed neuronal death. For autophagy, ubiquitination of the cargo is the prerequisite for its degradation. p62/SQSTM1, an adaptor protein, simultaneously binds to ubiquitinated cargo and LC3, which enables the degradation of itself and cargo in the lysosome [48]. Since the induction of autophagy is usually accompanied by a decrease in p62, and accumulation of p62 occurs after autophagy is inhibited, p62 may be used to monitor autophagic flux [49]. We found that OGD/R reduced the abundance of p62. Unexpectedly, treatment with ZD7288 at 10 μmol/L did not change the expression of p62 compared with OGD/R alone. If ZD7288 had no effect on autophagosome formation, its correction of excessive autophagy might be due to the acceleration of autophagic degradation, and would decrease p62 expression, which contradicts our results. Sahani *et al*. have reported that the expression level of p62 depends on autophagic degradation, changes in transcription and translation, and the availability of lysosome-derived amino-acids, so the p62 level is not always inversely related to autophagic activity [50]. Thus, p62 cannot be used as an indicator of the effect of ZD7288 on autophagic flux.

The accumulation of autophagic vacuoles can have three sources, selective impairment of autophagosome–lysosome fusion, dysfunction of lysosomal proteolysis, or a decrease in the number of lysosomes. The most widely used chemicals that inhibit the last stage of autophagy are CQ, bafilomycin A1 (BafA1), and lysosomal protease inhibitor cocktails. BafA1 inhibits the degradative capacity of lysosomes by decreasing their acidity, but it also impairs fusion between autophagosomes and lysosomes [51, 52]. In contrast, Mauthe *et al*. have reported that CQ inhibits autophagic flux by decreasing autophagosome–lysosome fusion, but not the degradative capacity of lysosomes [53, 54]. So we used CQ to evaluate whether ZD7288 affects the fusion of autophagosomes and lysosomes. Our results showed that, in the presence of CQ, treatment with ZD7288 at 10 μmol/L in the OGD/R group did not change the excessive expression of LC3-II compared with OGD/R alone, which meant that disrupting the fusion of autophagosomes and lysosomes cancelled the effect of ZD7288. On the other hand, if ZD7288 promoted or inhibited autophagosome formation, the accumulation of LC3-II in the OGD/R+CQ+ZD7288 group would be increased or decreased compared with the OGD/R+CQ group. Furthermore, we examined the expression of LAMP-1 and cathepsin D by Western blot analysis. Consistent with previous research [55], we found that the expression of LAMP-1 did not change significantly after ischemia, but cathepsin D expression was remarkably increased. However, treatment with ZD7288 at 10 μmol/L in the OGD/R group did not change the expression of LAMP-1 and cathepsin D, suggesting that ZD7288 had no effect on lysosomal proteolysis and the number of lysosomes. Thus, our findings demonstrated that ZD7288 has no effect on autophagosome formation, and it provides neuroprotection against OGD/R by accelerating autophagic degradation, which might be attributed to the promotion of autophagosome and lysosome fusion.

Studies have reported that ZD7288 may interact with Na^+^ and Ca^2+^ channels [56, 57], and is not an isoform-selective blocker of the HCN channel [58]. In the present study, we found that OGD/R injury resulted in significantly increased HCN2 channels but not HCN1 channels in HT22 neurons. shRNA knockdown of HCN2 channels increased neuronal survival in the OGD/R model of HT22 neurons. However, CQ eliminated the neuroprotective effect of HCN2-shRNA. We further demonstrated that, in OGD/R injury, HCN2-shRNA significantly reduced the accumulation of LC3-II in neurons. However, in the presence of CQ, HCN2-shRNA failed to reduce this accumulation, and the level of LC3-II did not significantly differ from OGD/R+CQ. Similarly, if HCN2-shRNA promoted or inhibited autophagosome formation, the accumulation of LC3-II would be increased or decreased compared with the OGD/R+CQ group. Thus, our findings demonstrated that knockdown of HCN2 channels also had no effect on autophagosome formation; it corrected the excessive autophagy induced by OGD/R injury due to the acceleration of autophagic degradation that might be attributed to the promotion of autophagosome and lysosome fusion. In HCN2-shRNA-transfected HT22 neurons, ZD7288 did not further reduce LC3-II accumulation, which suggested that the neuroprotection against OGD/R injury by ZD7288 is due to blockade of HCN2 channels. In order to further evaluate the effect of HCN2-shRNA on the fusion of autophagosomes and lysosomes, we performed immunohistochemical co-staining with antibodies against LC3 and LAMP-1 in HT22 neurons. We found that the percentage of LC3-positive puncta that co-localized with LAMP-1-positive lysosomes decreased in Con-shRNA-transfected neurons exposed to OGD/R or CQ. In HCN2-shRNA-transfected neurons, the percentage of LC3-positive puncta that co-localized with LAMP-1-positive lysosomes increased under OGD/R, but the percentage was significantly decreased by the addition of CQ to HCN2-shRNA-transfected neurons. Our results suggested that HCN2-shRNA promotes the fusion of autophagosomes and lysosomes in HT22 neurons.

Next, we performed experiments in the rat TGCI model to validate our findings. Consistent with the above results, shRNA knockdown of HCN2 channels markedly diminished TGCI-induced neuronal loss in the hippocampal CA1 area and ameliorated the cognitive impairment in rats. Besides, in the presence of CQ, HCN2-shRNA did not offer neuroprotection against TGCI injury. Furthermore, in the TGCI model, HCN2-shRNA also significantly reduced the accumulation of LC3-II in neurons, and CQ abolished the effects of HCN2-shRNA. These results further indicated that disrupting autophagic degradation eliminates the neuroprotective effect of HCN2-shRNA on the TGCI model in rats.

Although the present study has yielded some preliminary findings, several limitations to this pilot study need to be acknowledged. First, although HT22 cells are widely used, the data obtained from this cell line might differ from primary hippocampal neurons and animal studies. Second, our results indicated that ZD7288 had no significant effect on cell viability. In addition, ZD7288 (OGD/R+ZD7288) had no significant effect on the expression of autophagy-related proteins. So we did not further measure the effect of ZD7288 alone on the expression of autophagy-associated proteins in HT22 neurons. Third, the mechanisms underlying the OGD/R-induced increase of HCN2 channels in HT22 cells need further evaluation. Fourth, it is necessary to find an appropriate method to further investigate the effect of HCN2 channels on autophagosome and lysosome fusion in the case of cerebral ischemia *in vivo*. Fifth, we cannot rule out the possibility that ZD7288 regulates autophagy *via* other HCN channels, Na^+^ or Ca^2+^ channels. Further investigations are under way in our laboratory.

In conclusion, our present results demonstrated that blockade of HCN2 channels provides neuroprotection against OGD/R and TGCI injury by accelerating autophagic degradation, which might be attributed to the promotion of autophagosome and lysosome fusion in hippocampal neurons.
